# Magnetoresponsive Fiber-Reinforced Periodic Impedance-Gradient Absorber: Design and Microwave Absorption Performance

**DOI:** 10.3390/nano16010042

**Published:** 2025-12-29

**Authors:** Yuan Liang, Wei Chen, Shude Gu, Xu Ding, Yuping Duan

**Affiliations:** 1 International School of Information Science and Engineering, Dalian University of Technology, Dalian 116085, China; 1980002155@mail.dlut.edu.cn; 2 School of Materials Science and Engineering, Dalian University of Technology, Dalian 116085, China; chen93456@mail.dlut.edu.cn (W.C.); mate_gusd@mail.dlut.edu.cn (S.G.); 3011461348@mail.dlut.edu.cn (X.D.)

**Keywords:** dual-gradient stealth, broadband metamaterials, impedance gradient, multiresponse

## Abstract

In recent years, achieving ultra-wideband electromagnetic absorption has emerged as a critical challenge in confronting advanced broadband electromagnetic detection technologies. This capability is essential for effectively countering sophisticated radar systems. In this study, we present a novel multilayer metamaterial absorber that integrates an FR4 transmission layer, a periodic gradient dielectric structure designed for resonant impedance matching, and a magnetic skin layer for enhanced energy dissipation. By employing asymptotic gradients in both structure and composition, this design achieves dual-gradient electromagnetic parameter modulation, enabling efficient absorption across the X, Ku, and K bands (8.6–26.4 GHz) with a total thickness of 3.5 mm (effective thickness: 2 mm) and a density that is one-third that of conventional magnetic metamaterials. The proposed absorber demonstrates polarization insensitivity, stability across wide incident angles (up to 60°), and an absorption efficiency of 94%, as confirmed by full-wave simulations and experimental validation. Moreover, the fiber-reinforced hierarchical structure addresses the traditional trade-off between broadband absorption performance and mechanical load-bearing capacity.

## 1. Introduction

With the advancement of military weaponry, civilian electronic devices, stealth technologies, and radar detection systems, the investigation of their electromagnetic stealth and mechanical performance has garnered significant research attention [1,2,3,4,5,6]. Traditionally, wave-absorbing materials are fabricated by combining an insulating substrate with an absorbing medium, where the electromagnetic response is determined by the intrinsic absorption characteristics of the absorber and its dispersion behavior within the substrate [7,8,9]. A wide range of materials have been employed as absorbers, including carbon nanotubes (CNTs), carbon fibers, graphene, and reduced graphene oxide (RGO) [10,11,12,13,14]. Among them, carbon nanomaterials—such as one-dimensional CNTs and two-dimensional graphene nanosheets (GNs)—have attracted particular interest due to their distinctive chemical structures and tunable electrical properties. In recent efforts, researchers have incorporated magnetic components such as ferrites into CNTs and GNs to simultaneously leverage electric and magnetic losses, thereby enabling modulation of both the complex dielectric constant and complex permeability [15,16,17]. Achieving superior wave-absorbing performance requires not only high microwave attenuation but also effective impedance matching. However, due to the inherent resonance thickness dependence, merely adjusting the composition ratio of powdered absorbers within the matrix imposes limitations on enhancing broadband absorption capability [18,19].

Therefore, to enhance the overall performance of microwave absorbers—particularly in achieving both wide absorption bandwidth and high attenuation efficiency—it is essential to adopt advanced structural strategies, such as multilayer gradient configurations and meta-structural designs. By selecting appropriate multilayer gradient electromagnetic parameters, the wave absorption performance can be significantly optimized [20,21]. Such design strategies greatly enhance impedance matching and effectively broaden the absorption bandwidth of the material [22,23,24,25,26,27,28,29]. Nevertheless, absorbers based on single-loss mechanisms often struggle to realize broadband absorption, which has led to the development of metamaterial structures as an innovative solution for improving wideband microwave absorption performance [30,31,32]. However, metamaterial absorbers without sufficient mechanical robustness remain limited in their practical application. Moreover, a well-known trade-off exists between electromagnetic performance and mechanical strength, where improvements in one typically come at the expense of the other [33]. Several studies have demonstrated that enhancing mechanical strength may require compromising on absorption bandwidth [34,35,36]. Thus, a novel design strategy is urgently needed to address this dilemma. In parallel, recent studies have explored the principles of electromagnetic coupling and impedance matching, which offer valuable theoretical guidance for the structural and functional design of advanced metamaterials [37,38,39,40].

Conventional wave-absorbing materials primarily rely on adjusting absorber composition; however, due to the inherent resonance thickness dependence, it remains challenging to simultaneously achieve broadband absorption and satisfactory mechanical performance. Additionally, high-frequency absorption is often hindered by impedance mismatches resulting from a single loss mechanism—either dielectric or magnetic. In response to these limitations, we propose a fiber-reinforced periodic gradient absorber (ASM) that integrates the following components:

1. An FR4 transmission layer for impedance matching,
2. A dielectric meta-structure to induce multi-reflection and resonance,
3. A magnetic skin layer (CIP/MWCNTs/PU) for electromagnetic energy dissipation.

This structure achieves asymptotic dual-gradient regulation of electromagnetic parameters through a multilayer gradient design: the FR4 wave-transparent layer improves incident wave transmission, the periodic gradient dielectric structure enhances impedance matching via multiple internal reflections, and the magnetic skin layer provides strong magnetic loss. The introduction of a fiber-reinforced design, using glass fiber cloth as a structural support layer, not only strengthens mechanical load-bearing capacity but also facilitates the orderly dispersion of absorbing components through surface micro-topography, thereby alleviating interfacial impedance mismatches. A tower-shaped superstructure is incorporated to form a periodic gradient architecture with controlled lateral prismatic angles (optimal at 70°), enabling efficient absorption of obliquely incident waves while reducing total thickness (effective thickness: only 2 mm).

Through this design, the ASM metamaterial achieves ultra-broadband absorption, with an effective absorption bandwidth spanning 8.6–26.4 GHz (covering the X, Ku, and K bands), while maintaining a density just one-third that of conventional magnetic superstructures—thereby overcoming traditional limitations of excessive thickness (>5 mm) and high density. The structure is polarization-insensitive due to its quadruple rotational symmetry, maintaining TE/TM polarization absorptivity above 94% across incidence angles from 0° to 60°. Additionally, the far-field radar cross-section (RCS) is markedly reduced compared to a perfect electric conductor (PEC), with peak scattering attenuation reaching −22 dB at 22.4 GHz. The fiber-reinforced construction further imparts excellent mechanical robustness, making the material highly suitable for aerospace stealth applications.

## 2. Experimental Procedure and Design

### 2.1. Preparation of Materials

Carbonyl iron powder (CIP) was purchased from China Shanxi Xinghua Chemical Co., polyurethane (PU) resin was supplied by China Shanghai Synthetic Polymers Co., Ltd., fiberglass cloth was obtained from China Anhui Hannao New Material Co., Ltd., and FR4 boards were sourced from the China Anhui Hannao New Material Co., Ltd. All materials were commercially available and used directly without further purification.

To characterize the electromagnetic parameters, mixtures with varying mass ratios were prepared and cured in specialized molds to form coaxial ring-shaped specimens with an outer diameter of 7 mm and an inner diameter of 3.04 mm. The complex permittivity and permeability of the composites were measured using the coaxial reflection/transmission method over a frequency range of 2–18 GHz, employing an Agilent 8722 ES vector network analyzer (Agilent Technologies, Santa Clara, CA, USA).

In parallel, the S-parameters, electromagnetic field distribution, polarization sensitivity, and radar cross-section (RCS) of the samples were simulated and analyzed using the Ansys Electronics High Frequency Structure Simulator (HFSS, Ansys Inc., Canonsburg, PA, USA). Additionally, experimental validation of the samples’ reflection loss was conducted in a microwave anechoic chamber using the standard arch measurement method.

Supporting Information Note S2 details the characterization and performance testing of raw materials, along with simulation methodologies.

Supporting Information Note S3 details the software and specific settings required for electromagnetic simulation in this study.

Supporting Information Note S4 presents the electromagnetic parameters of the raw materials in this study at frequencies ranging from 1 to 18 GHz.

Supporting Information Note S6 lists the surface density and bulk density of the raw materials used.

### 2.2. Experimental Preparation

In this study, polyurethane (PU) resin was selected as the matrix material due to its excellent fluidity and flexibility, which confer notable advantages when blended with electromagnetic absorbers. Multi-walled carbon nanotubes (MWCNTs) were employed as the dielectric absorber for the outer layer owing to their outstanding dielectric properties. However, the inherent poor dispersion of MWCNTs necessitated a dispersion treatment. To this end, a low-ratio mixture of polyvinylpyrrolidone (PVP):MWCNTs in water was utilized for processing. Carbonyl iron powder (CIP) was chosen as the magnetic absorber because of its uniform spherical morphology and smooth surface, which contribute to its superior magnetic loss performance.

Due to the weak interfacial adhesion between fiberglass cloth and the PU matrix, the surface of the fiberglass was modified using a silane coupling agent (KH550). During the modification process, the glass fibers were immersed in a solution of specific concentration for 30 min, followed by drying at 80 °C for 2 h. Given the tendency of MWCNTs to agglomerate as a result of strong van der Waals interactions, which can inhibit their interfacial polarization and conductivity, a dispersion system composed of PVP:MWCNTs:H_2_O in a mass ratio of 1.25:5:93.75 was prepared. This mixture was subjected to magnetic stirring and ultrasonic treatment, and the resulting MWCNTs were collected after air drying.

The CIP/PU and MWCNTs/PU wave-absorbing composites were fabricated using the coaxial testing method. Specific volume fractions of CIP and MWCNTs were added to the resin matrix and thoroughly mixed to obtain a homogeneous system. A curing agent was subsequently introduced and mixed uniformly for 30 min. The resulting mixture was then degassed under vacuum for 30 min and cast into flexible silicone molds, followed by room temperature curing for 12 h to produce specimens for electromagnetic parameter testing. The structural parameters of the fabricated samples were set as follows: h1 = 0.5 mm, h2 = 2 mm, h3 = 1 mm, a1 = 0.9 mm, and a2 = 2 mm.

**Figure 1:**
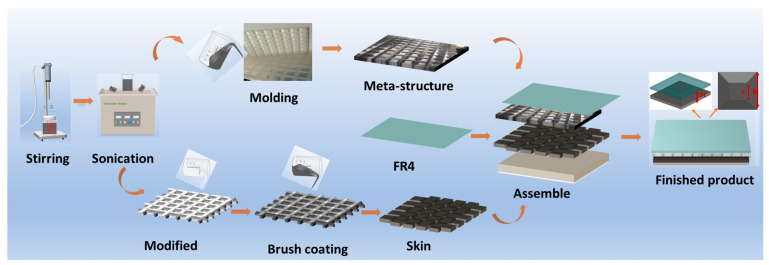
Preparation process of ASM composites.

To validate and characterize the reflection loss of the designed gradient CIP/MWCNTs/PU metamaterials within the 2–18 GHz frequency range, a structured composite plate with dimensions of 200 mm × 200 mm × 3 mm was fabricated using a two-step molding process. The detailed fabrication and impedance gradient preparation flow are illustrated in Figure 1.

A positive mold was first fabricated via 3D printing, followed by the preparation of a negative mold using silicone. Polyurethane (PU) was mixed with varying concentrations of MWCNTs through a combined process of magnetic stirring and ultrasonication, each for 8 min, repeated three times to ensure uniform dispersion. The CIP/PU mixtures were prepared using the same procedure. A predefined volume of the MWCNTs/PU mixture was poured into the silicone mold, upon which a layer of fiber cloth was laid. Subsequently, the CIP/PU wave-absorbing adhesive was uniformly brushed onto the surface. The coating and brushing process was repeated twice, after which the entire structure was allowed to cure at room temperature for 12 h.

Upon completion of curing and demolding, the resulting gradient CIP/MWCNTs/PU metamaterial—hereafter referred to as SAM—was bonded with an FR4 wave-transmissive layer to form the final integrated FR4-SAM structure, denoted as ASM.

The periodic gradient dielectric structure within the absorber adopts a hexagonal lattice arrangement composed of unit cells with 10 mm edge lengths. Each unit cell exhibits a truncated pyramid geometry, with the base width linearly decreasing from a1 = 2.0 mm at the bottom to a2 = 0.9 mm at the top, thereby forming a sidewall gradient angle of 70° relative to the normal direction (Figure 1). The height of each unit is fixed at h2 = 2.0 mm, and the spacing between adjacent units is d = 1.5 mm.

The gradient in electromagnetic parameters is achieved through spatial variation in component mass ratios across the thickness. Specifically, the MWCNTs concentration in the dielectric layer increases linearly from 0.5% (SP1) at the outer surface to 1.0% (SP2) toward the interior. Conversely, the CIP concentration in the magnetic skin layer decreases from 83.3% (DS1) to 66.6% (DS2) from bottom to top. This dual-gradient design enables effective impedance matching while simultaneously promoting multi-scale electromagnetic energy dissipation.

The microstructure of the CIP/MWCNTs/PU composites was examined using a field emission scanning electron microscope (SEM, model JSM-7610FPlus, JEOL Ltd., Tokyo, Japan) To evaluate the electromagnetic performance, the complex permeability and permittivity of the composites were measured using the coaxial transmission/reflection method. The tests were conducted over a frequency range of 2 to 18 gigahertz using a vector network analyzer (VNA, model N5234A, Keysight Technologies, Santa Rosa, CA, USA), with samples prepared in a ring geometry having an outer diameter of 7 mm, an inner diameter of 3 mm, and a thickness of 3 mm.

## 3. Results and Discussion

### 3.1. Optimization of Electromagnetic Parameters and Structural Geometry

This section investigates the electromagnetic absorption loss mechanisms in the fiber-reinforced periodic gradient absorbers, which stem from the synergistic contributions of magnetic and dielectric losses and are closely tied to impedance matching behavior. The outer dielectric structure exhibits multiple internal reflections, allowing the SP1 configuration to optimize impedance matching. Although SP1 offers superior wave transmittance, its intrinsic loss capability is weaker than that of SP2. Here, SP1 and SP2 refer to composite systems comprising MWCNTs and polyurethane in mass ratios of 0.5:99.5 and 1:99, respectively. Similarly, DS1 and DS2 refer to carbonyl iron powder (SCIP) and PU mixed at ratios of 83.3:16.6 and 66.6:33.3, respectively.

For the magnetic skin layer, DS1 demonstrates stronger energy dissipation than DS2, largely due to its thinner configuration, which promotes enhanced magneto-electric coupling. Therefore, considering both impedance matching and loss efficiency, the combination of SP1 in the dielectric gradient structure and DS1 in the magnetic skin structure is the most favorable configuration for achieving optimal microwave absorption performance.

**Figure 2:**
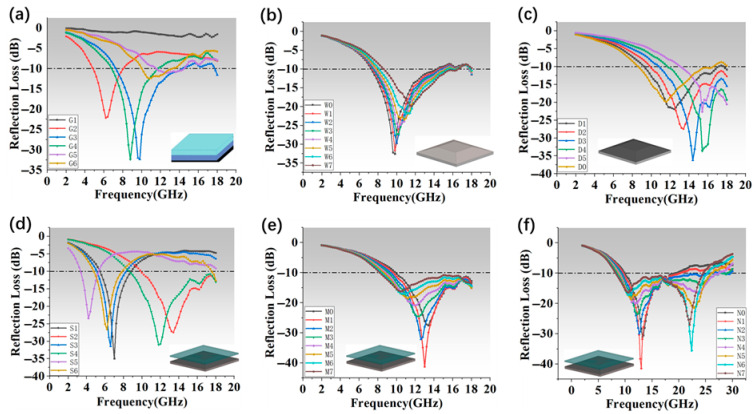
(**a**) Absorption performance of G1–G6 with different electromagnetic parameters. (**b**) W0–W7 absorption performance for different prism and normal angles. (**c**) D0–D5 absorption performance for different fiber cloth layers. (**d**) S1–S6 absorption performance for different meta-structure height difference. (**e**) M0–M7 are the absorption properties of FR4 with different thicknesses. (**f**) Absorption properties of FR4 with different thicknesses derived from the first-order Debye equation.

In the preliminary design phase, a range of electromagnetic parameter combinations was developed by systematically adjusting component concentration gradients. This yielded six representative parameter groups: SP1 + SP2, DS1 + DS2, SP1 + DS1, SP1 + DS2, SP2 + DS1, and SP2 + DS2, designated as G1 through G6. As illustrated in Figure 2a, the dielectric-only group exhibited weak energy loss and a singular loss mechanism, resulting in limited absorption performance. In contrast, the magnetic-only group performed well in the low-frequency region but lacked effectiveness at higher frequencies. Among the remaining four hybrid combinations, G3—consisting of SP1 and DS1—achieved the best overall microwave absorption performance.

The skin layers were fabricated by stacking and curing the prepared prepregs. Within the skin structure, the embedded fiber cloth serves as the impedance matching layer, while the applied wave-absorbing adhesive acts as the functional coating responsible for electromagnetic absorption. The fiber cloth not only reinforces the structural integrity but also supports layered architecture, thereby enhancing the mechanical strength and facilitating multiple internal wave reflections and absorption.

By configuring the outer dielectric layer as a dedicated matching layer and isolating the magnetic loss behavior of the inner layer, the power dissipation of each component, as well as the transmission characteristics of the outer dielectric structure, can be independently evaluated. Among the reference groups, G1 (optimized for dielectric gradient) and G2 (optimized for magnetic gradient) were selected for comparative analysis. G1 displayed poor absorption due to its low dielectric content, resulting in an ineffective loss mechanism. G2, on the other hand, failed to establish sufficient magneto-electric coupling at high frequencies, leading to significant impedance mismatch.

Through further optimization of the concentration gradients in groups G3 through G6, it was determined that SP1 is the preferred outer layer for enhancing magneto-electric synergy with the underlying carbonyl iron layer, in contrast to SP2. The relatively low concentration of MWCNTs in SP1 leads to a less developed conductive network, which improves transmission while reducing reflectivity—an effect beneficial for matching. In comparison, the bottom layer configured with DS2, which contains a higher fraction of carbonyl iron, substantially improves microwave magnetic loss capability. Notably, although increased carbonyl iron content may contribute to higher reflectivity, its adverse effects are mitigated by the enhanced intrinsic magnetic loss, thus supporting the overall absorption efficiency of the structure.

Among the six configurations, G3 was selected as the optimal combination for impedance matching, yielding superior broadband absorption performance. To further enhance this effect, the dielectric coating layer was designed as a gradient meta-structure with its performance tuned by varying the prism inclination angle. The meta-structure height was fixed at 2 mm, while the inclination angle between the prism’s side and the vertical axis was adjusted systematically. As shown in Figure 2b, increasing the angle led to a progressive reduction in the overall volume of the meta-structure; nevertheless, the effective absorption bandwidth was significantly broadened, despite a slight decrease in peak absorption. At an optimal angle of 70 degrees, the structure’s volume was reduced to just one-quarter of an equivalent full-thickness layer, yet absorption performance was substantially improved. The “tower-like” morphology of this configuration proved particularly effective for capturing obliquely incident waves. The inclined gradient sides promoted multiple internal reflections, enhancing the electromagnetic wave attenuation through angular scattering paths. This multi-scattering mechanism compensates for reduced material thickness by increasing effective propagation length and dissipative interactions.

To investigate the effect of the fiber-reinforced structure, a series of samples with varying numbers of fiber cloth layers were fabricated. As illustrated in Figure 2c, increasing the number of fiber layers led to a progressive enhancement of the wave absorption modulation effect. Both the absorption peak frequency and magnitude were observed to rise with added layers. Notably, the incorporation of two layers of fiber cloth provided a balanced structure: it ensured that the high-frequency absorption peak remained within the effective attenuation range while minimizing undesired low-frequency shifts.

In order to further examine the interplay between meta-structure and magnetic skin thickness on absorption performance, six sample groups (S1–S6) were designed with fixed total thicknesses of 3, 4, and 5 mm. For the 3 mm group, S1 employed a meta-structure and skin thickness of 1 mm and 2 mm, respectively, while S2 used the reverse configuration. For the 4 mm group, S3 featured 2 mm meta-structure and 1 mm skin, while S4 had 1 mm and 2 mm, respectively. Similarly, S5 and S6 in the 5 mm group featured meta-structure/skin combinations of 3 mm/2 mm and 2 mm/3 mm, respectively.

The absorption spectra of S1–S6, as shown in Figure 2d, revealed that samples with thinner meta-structures and thicker skin layers exhibited red-shifted absorption peaks and poor impedance matching at high frequencies. Conversely, increasing the meta-structure thickness relative to the skin led to a blue shift in the absorption peak and improved high-frequency matching. S2 and S4 demonstrated this effect clearly, confirming that a thicker meta-structure with a thinner magnetic skin benefits high-frequency impedance alignment. In contrast, S1 and S3, despite having good low-frequency coverage, suffered in the high-frequency range due to impedance mismatch. Sample S6, with its excessively thick meta-structure, exhibited degraded wave transmission and further mismatch. S5 showed the poorest performance overall, as the combined effects of high impedance mismatch and low transmission efficiency limited both absorption bandwidth and peak strength. These findings underscore the need to consider not only intrinsic material losses but also wave transmission and impedance matching in absorber structural design.

Based on a comprehensive analysis, sample S2—with a total thickness of 3 mm—was selected as the representative configuration for this study, balancing compact structure with optimal impedance matching. As shown in Figure 2e, simulations of structures N0 through N7 revealed that increasing the FR4 layer thickness caused a noticeable shift in absorption peaks toward higher frequencies. Notably, a secondary peak begins to emerge around 18 GHz, suggesting that fine-tuning of FR4 thickness could further expand the absorption bandwidth into the 18–30 GHz range.

Thus, the dielectric constant was derived using the first-order Debye equation. From this derivation, it is evident that the first-order Debye model effectively characterizes the frequency response behavior of dielectric materials, particularly describing the dielectric relaxation phenomena of polar molecules. The corresponding expression for the complex dielectric constant provides a theoretical basis for understanding the variation in permittivity across different frequencies.

Based on this model, the microwave absorption performance of FR4 layers with varying thicknesses in the 2–30 GHz range was simulated using HFSS, as illustrated in Figure 2f (N0–N7). The results show a progressive shift in the absorption peaks toward lower frequencies as the FR4 thickness increases from 0.1 mm to 0.7 mm. This redshift is attributed to enhanced impedance matching and improved transmission characteristics of FR4, which also contributes to superior absorption performance in the high-frequency region.

Integrating the above findings, the FR4 wave-transmissive layer plays a dual role in wavelength matching and phase modulation. Specifically, the inclusion of a 0.5 mm FR4 layer optimizes the transmission of incident electromagnetic waves and facilitates absorption peak shifts to lower frequencies. For instance, the N5 configuration exhibits dual-peak absorption at 11.2 GHz and 22.8 GHz, effectively broadening the total absorption bandwidth to 17.8 GHz.

Based on the above study, a dual-gradient electromagnetic parameter design was developed to optimize both impedance matching and energy attenuation capability. The gradual modulation of the dielectric constant and magnetic permeability is achieved through the combined effect of the periodic gradient dielectric structure (SP1–SP2) and the magnetic surface layer (DS1–DS2).

In this configuration, the low concentration of MWCNTs (0.5%) in the outer layer improves electromagnetic wave transmittance, while the high concentration of CIP (83.3%) in the inner layer enhances magnetic loss, thereby resolving the impedance mismatch problem commonly associated with single-mechanism loss systems.

Furthermore, the dual-gradient structure enables significant volume reduction while maintaining effective broadband absorption. Specifically, a tower-type superstructure unit with a prism side inclination angle of 70° is adopted, resulting in a unit volume that is only one-quarter that of a conventional homogeneous structure. Simultaneously, the inclined geometry extends the propagation path of electromagnetic waves through multiple internal reflections—up to 1.7 times the wavelength—leading to more than 85% energy attenuation and ensuring effective absorption across the X, Ku, and K bands (8.6–26.4 GHz). The design also introduces a fiber-reinforced structure as a novel element.

Mechanical–electromagnetic synergistic design: Glass fiber cloth is utilized as a structural support layer, and the micron-scale surface height variation promotes the orderly dispersion of the absorber. This not only enhances the mechanical strength of the structure—making it suitable for aerospace load-bearing applications—but also helps mitigate interfacial impedance mismatch.

Additionally, the use of multilayer fiber cloth plays a regulatory role in tuning the absorption frequency bandwidth. Specifically, in the ASM structure, the introduction of a double-layer fiber cloth enhances the stability of high-frequency absorption peaks while preventing shifts in the low-frequency peak position, thereby achieving an optimal balance between broadband absorption performance and mechanical robustness.

### 3.2. Performance Validation

**Figure 3:**
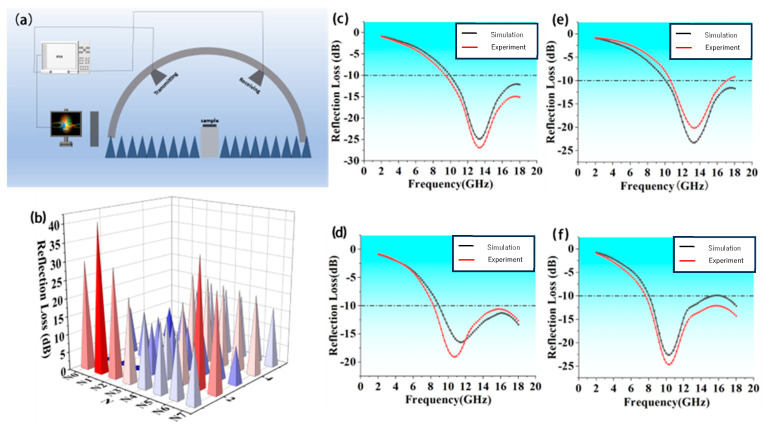
(**a**). Test Scenario Diagram. (**b**) Rlmin (dB)1, Rlmin (dB)2, peak frequency (GHz)1, peak frequency (GHz)2 and EAB (dB) of FR4 with different thicknesses from N0 to N7 at 2–30 GHz. (**c**) Simulated and measured absorption performance of SP1 + DS1 without FR4. (**e**) Simulated and measured absorption performance of SP1 + DS1 without FR4. (**d**) Simulated and measured absorption performance of SP1 + DS1 with FR4. (**f**) Simulated and measured attenuation of SP2 + DS1 with FR4.

(Figure 3c–f) display the measured and simulated performance of SP + DS1 absorbing structures without FR4, SP2 + DS1 absorbing structures without FR4, SP1 + DS1 absorbing structures with FR4, and SP2 + DS1 absorbing structures with FR4, respectively. The reflection loss of the samples was measured in a microwave anechoic chamber, and the tests were conducted over the 2–18 GHz frequency range using the arched method, as depicted in Figure 3c. The effective absorption bandwidth of these structures is closely related to their peak absorption values. According to established literature on wave absorption, the measured absorption performance of the structures is generally weaker than that of the simulated structures due to errors arising from CIP settling and the mold preparation process. However, it is noteworthy that the measured performance of the structures in (Figure 3c,d) exceeds that of the simulated structures, while the measured performance of the structures in Figure 3e,f is inferior to the simulated counterparts. This discrepancy can be attributed to the bottom layer of the masks, which consists of multilayer fiber cloth brushed and coated with curing adhesive.

The bottom layer consists of a multilayer fiber fabric that has been brushed, coated with wave-absorbing adhesive, and subsequently cured. In the simulated structure, the fiber cloth within the skin layer functions as an impedance matching layer, while the wave-absorbing adhesive serves as a coating that operates as the primary absorption layer.

The fiber cloth plays a dual role as both a structural support material and a layered template, significantly enhancing the composite’s mechanical strength and electromagnetic absorption performance. Notably, the surface of the glass fiber cloth presents a distinctive micron-scale height variation and inter-fiber gaps, which promote the orderly distribution of absorber particles across the fiber layer.

This two-dimensional fiber cloth templating strategy effectively mitigates the negative effects of CIP sedimentation and fabrication inconsistencies associated with mold preparation, thereby contributing to improved reliability and uniformity in wave-absorbing performance.

The performance degradation observed in Figure 3e,f is attributed to the inherent difficulties in fabricating meta-structures with high concentrations of MWCNTs, as compared to those using lower concentrations within the matrix. During the mold preparation process, partial gelation of MWCNTs may occur, leading to the formation of a localized conductive network that hinders the transmission of electromagnetic waves.

This electromagnetic shielding effect counteracts, to some extent, the beneficial influence of the two-dimensional fiber cloth template, ultimately causing the measured absorption performance to fall below the levels predicted by simulation.

Figure 3b presents the simulated absorption performance of the N0–N7 structural configurations across the 2–30 GHz frequency range. In this context, RLmin (dB)1 and RLmin (dB)2 represent the first and second effective absorption peaks, respectively, while Peak Frequency 1 and Peak Frequency 2 denote the corresponding resonance frequencies. The effective absorption bandwidth (EAB) refers to the total frequency range over which the absorption exceeds the effective threshold.

These N0–N7 structures are designed as superstructures incorporating FR4 layers of varying thicknesses ranging from 0 to 0.7 mm. Among them, the N5 configuration demonstrates an EAB of 17.8 GHz, with the first and second absorption peak values reaching −19 dB and −22 dB at 11.2 GHz and 22.8 GHz, respectively.

Although N5’s total EAB is slightly narrower than that of N3 (19.8 GHz), it exhibits a well-defined dual-peak absorption profile, with strong performance extending into the lower portion of the K-band. The ASM structure—featuring a 0.5 mm FR4 layer as its outer superstructural component—not only benefits from intrinsic and structural loss mechanisms but also warrants deeper investigation into the underlying physical principles contributing to its exceptional absorption performance.

### 3.3. Electric/Magnetic Field Distribution and Loss Mechanisms

**Figure 4:**
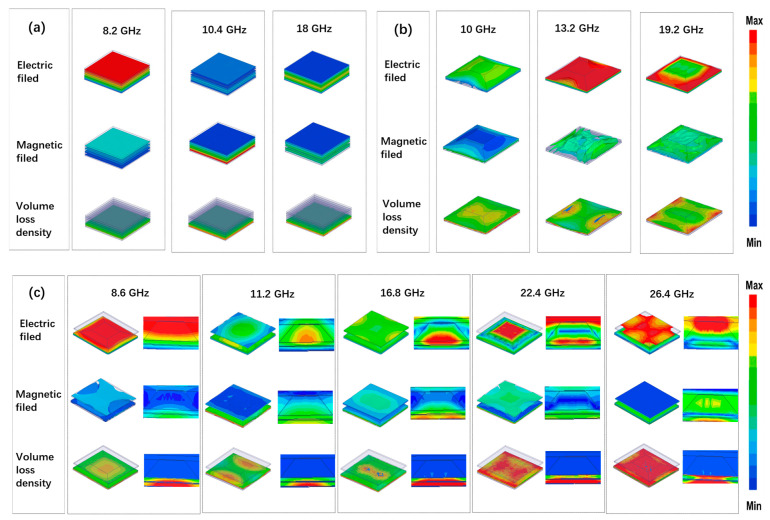
(**a**) Simulated electromagnetic field distribution and volume loss density in a structure with heterogeneous electromagnetic parameters. (**b**) Simulation results of electromagnetic field intensity and corresponding power loss for the SAM structure. (**c**) Simulation results of electromagnetic field intensity and corresponding power loss for the ASM structure.

As shown in Figure 4a,b, with increasing frequency, the region of electromagnetic wave loss progressively shifts toward the interior of the material. Analysis of the electromagnetic field distribution in materials with uniform thickness reveals distinct variations in absorption behavior. The introduction of a heterogeneous interface leads to improved impedance matching performance.

The incorporation of a meta-structured layer introduces multiple internal reflections and distinctive transmission characteristics. These arise from the incomplete conductive network formed by carbon nanotubes within the resin matrix, in conjunction with the intrinsic geometric features of the meta-structure. Together, these effects result in enhanced absorption performance.

As frequency increases, the electric field begins to extend from the interior toward the exterior regions of the absorber. Notably, the strongest absorption is observed at 10.4 GHz, which is attributed to the synergistic effect of pronounced magnetic loss in the bottom layer and the heterogeneously distributed electromagnetic parameters in the outer dielectric layer.

The current wave-absorbing structure, comprising a combination of wave-transparent, dielectric, and magnetic components, exhibits more complex electromagnetic field evolution compared to conventional homogeneous absorbers. Relative to the ASM structure without the FR4 layer, the SAM demonstrates a gradual enhancement in electric field intensity as frequency increases, reaching its maximum at 13.2 GHz.

However, beyond this frequency, the electric field strength decreases progressively from the top surface toward the bottom layer, indicating a reduction in wave-absorbing performance. This degradation is primarily attributed to magneto-electric mismatch arising at higher frequencies.

Consequently, in the gradient structure, electromagnetic wave loss gradually shifts from the bottom layer toward the top as frequency increases. In the absence of an FR4 layer, the meta-structure exhibits poor impedance matching with incident electromagnetic fields at high frequencies, thereby inhibiting the effective penetration of waves into the structure.

As a result, both magnetic and overall power losses are less significant under conditions without FR4 compared to those with FR4. The relatively weak absorption observed at low frequencies is primarily attributed to the presence of dielectric components, which hinder the transmission of electromagnetic waves in the lower frequency range.

Within the frequency interval of 8.6 to 11.2 GHz, the electric field gradually attenuates. The initial peak in the external electromagnetic field is primarily driven by the combined effects of dielectric and magnetic losses. This coupling enhances impedance matching and facilitates deeper wave penetration into the material.

In contrast, the incorporation of the FR4 layer in the ASM structure results in an extended effective wavelength and causes a redshift in the absorption peak toward lower frequencies. As shown in Figure 4c, compared to the case without FR4, the electric field distribution at the first absorption peak demonstrates a pattern of initial attenuation followed by local enhancement. Specifically, the electric field initially diffuses and attenuates from the outer region toward the inner layer, followed by repeated localized enhancements—reflecting the progressive energy dissipation during inward field propagation.

Additionally, the introduction of layered metamaterial interfaces induces pronounced interfacial effects, which significantly contribute to electromagnetic energy dissipation and enhance the overall absorption capacity of the structure. Based on the above analysis, the ASM achieves a strong magneto-electric coupling effect: the bottom CIP/PU layer serves as the magnetic loss region, while the surface MWCNTs/PU layer functions as the dielectric loss region. These layers act synergistically, with magnetic losses dominating in the high-frequency range (>10 GHz), and dielectric losses prevailing in the low-frequency range (<10 GHz), thereby enabling highly efficient absorption across the full operating bandwidth.

This performance enhancement is primarily attributed to interfacial energy dissipation induced by the gradient structure. The heterogeneous interface initiates strong interfacial polarization effects, leading to stepwise attenuation of incident electromagnetic waves from the exterior to the interior of the absorber, as illustrated in Figure 4a–c. The observed absorption peak at 10.4 GHz results from the synergistic matching between magnetic loss and the spatial variation in electromagnetic parameters.

### 3.4. Phase Modulation and Interference Effects

The phase difference between reflected waves at the front and rear interfaces of the absorber plays a crucial role in determining electromagnetic wave reflection behavior. When the material thickness is equal to one-quarter of the incident wavelength, the reflected wave acquires a phase shift of 180 degrees (or π radians) relative to the incident wave upon reciprocal propagation.

This phase shift induces destructive interference between the forward and backward reflected waves, effectively canceling out the reflected signal and significantly reducing total reflection. In practice, the two key interfaces—the air–absorber boundary and the absorber–metal substrate interface—each contribute reflected waves, and the phase relationship between them directly governs the absorber’s overall effectiveness.

When the phase difference between the two reflected waves equals an odd multiple of half the wavelength, destructive interference occurs, significantly enhancing electromagnetic wave attenuation. In contrast, if the phase difference corresponds to an even multiple of half the wavelength, constructive interference takes place, thereby reducing the overall attenuation efficiency.

Understanding the influence of material properties and microstructural features on electromagnetic wave absorption is fundamental to analyzing attenuation behavior. Effective absorber design requires not only consideration of the material’s intrinsic absorption characteristics but also the integration of wave interference theory—particularly the quarter-wavelength principle—to optimize performance.

This involves tuning the phase relationship between the reflected waves at the air–absorber interface (denoted as Rf) and those at the absorber–metal backing interface (denoted as Rb), thereby enhancing destructive interference and minimizing overall reflection.

**Figure 5:**
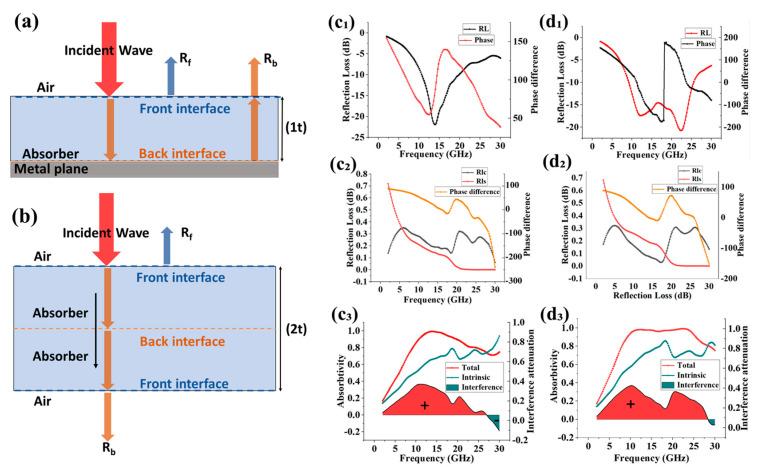
(**a**) Electromagnetic wave interface reflection model and (**b**) symmetry model. (**c_1_**,**d_1_**) RL and phase under the real model of SAM and ASM. (**c_2_**,**d_2_**) Amplitude and phase difference between the front and back interface waves under the symmetric model and SAM and ASM. (**c_3_**,**d_3_**) Intrinsic and interference attenuation characteristics of SAM and ASM.

As shown in Figure 5a,b, we analyzed the quantitative interference effects and intrinsic attenuation behavior of a 2.5 mm FR4-free SAM structure and a 3 mm ASM structure using wave interference theory and a symmetry-based modeling approach. As illustrated in Figure 5(c_1_), the reflection loss (RL) peak of the SAM coincides with a 180° phase shift, indicating a strong destructive interference condition. In contrast, Figure 5(d_1_) shows that the phase inflection point in the ASM—with the introduction of the FR4 layer—also corresponds to its absorption peak. However, the presence of a dual-phase deflection results in partial degradation of absorption performance.

Figure 5(c_2_,d_2_) indicates that within the frequency range where reflectivity is low, the front interface reflection wave Rf and the rear interface reflection wave Rb exhibit similar amplitudes. Furthermore, a phase difference of approximately 180° exists between them, equivalent to roughly half a wavelength. From the analysis in Figure 5(c_3_,d_3_), it is evident that the unique gradient structure of the SAM significantly enhances its intrinsic broadband absorption capability, while also suppressing the adverse effects of phase-length interference. Furthermore, the ASM, enhanced by the FR4 layer, increases the number of heterogeneous interfaces, thereby reinforcing the phase cancellation effect. This results in a marked improvement in broadband absorption efficiency.

These findings confirm that the ASM structure achieves a reduced RL slope in the high-frequency regime and realizes ultra-broadband absorption. This performance is not only attributed to the gradient structural design of the SAM and the impedance-matching benefits provided by the FR4 layer, but also validates the effectiveness of the theoretical framework and design methodology proposed in this study.

### 3.5. Radar Cross-Section and Polarization Characteristics

**Figure 6:**
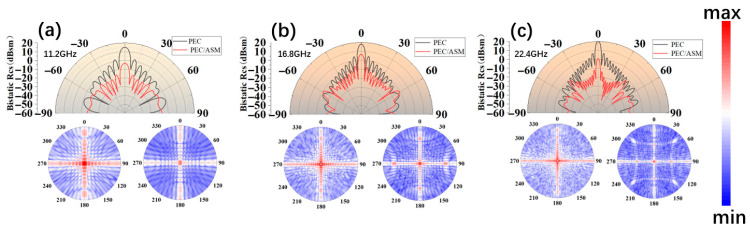
(**a**–**c**) Comparison of far-field radiograms and RCS after ASM stacking at 11.2 GHz, 16.8 GHz and 22.4 GHz, respectively.

The radar far-field scattering cross-section (RCS), which reflects polarization sensitivity, is a key metric for evaluating the performance of materials in electromagnetic environments. To investigate the stealth capability of the ASM structure, we conducted simulations using the HFSS platform. As shown in Figure 6a–c, an RCS model of the ASM was constructed, and far-field scattering analyses were performed at the absorption peaks under normal incidence. The results were compared against a reference perfect electric conductor (PEC).

The simulation reveals that the maximum scattering peaks at 11.2 GHz, 15.6 GHz, and 22.4 GHz are notably reduced for the ASM compared to the PEC, indicating a significant suppression of radar detectability and confirming the structure’s effective stealth performance.

**Figure 7:**
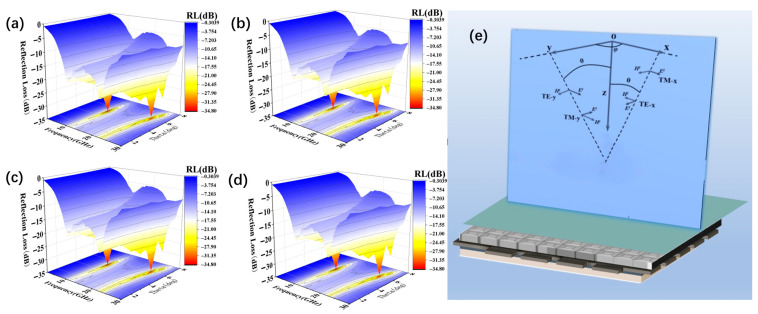
(**a**,**b**) Simulated absorption plots of the ASM at oblique incidence of the electromagnetic wave for the wave vectors of TE-polarized (TE-x) and TM-polarized (TM-x) waves incident along the XOZ plane. (**c**,**d**) Wave vectors of the ASM for the TE-polarized (TE-y) and TM-polarized (TM-y) waves incident along the YOZ plane. (**e**) Schematic of the linear polarization pattern of the ASM.

In addition, simulations were performed to assess the polarization sensitivity and angular stability of both ASM and SAM. As illustrated in Figure 7a–e, the absorption rates under transverse electric (TE) and transverse magnetic (TM) polarization modes were analyzed at varying polarization angles. The results demonstrate that the ASM maintains stable absorption performance as the polarization angle increases.

This polarization insensitivity is attributed to the structure’s fourfold rotational symmetry, which ensures consistent performance regardless of polarization direction. The dual-peak absorption response remains robust, particularly under TE polarization, where stable absorption is sustained at incident angles up to 60°.

Furthermore, the RCS reduction reaches 15 dB at 22.4 GHz (Figure 6), while polarization stability is preserved up to 60° (Figure 7). As shown in Table S1, the “fiber-reinforced periodic impedance gradient” strategy proposed in this study overcomes the inherent trade-offs between broadband performance, thin-layer thickness, wide-angle stability, and mechanical strength in conventional absorbers. This is achieved through the synergistic interaction of dual gradient parameters with structural design. While the fabrication process is relatively complex, it provides a critical solution for high-performance application scenarios.

## 4. Conclusions

In summary, this study demonstrates the synergistic optimization of broadband electromagnetic absorption, mechanical load-bearing capacity, and low radar cross-section (RCS) through the integration of structural gradient design, advanced material composition, and process innovation—offering a novel approach for next-generation aerospace stealth materials. By applying the principles of impedance matching and multi-scale structural engineering, the proposed design achieves simultaneous enhancement of ultra-wideband absorption and mechanical robustness. The key innovation lies in the strategic integration of a periodic gradient dielectric superstructure, a fiber-reinforced layer, and a magnetic loss skin. This combination effectively overcomes the limitations of conventional wave-absorbing materials, such as narrow absorption bandwidth and excessive thickness, thereby providing a lightweight and high-performance solution tailored for aerospace stealth applications.

## Data Availability

The original contributions presented in this study are included in the article/Supplementary Material. Further inquiries can be directed to the corresponding authors.

## Supplementary Materials

The following supporting information can be downloaded at: https://www.mdpi.com/article/10.3390/nano16010042/s1. Reference [41] is cited in the supplementary materials.
