# Supplementary material for: Magnetoresponsive Fiber-Reinforced Periodic Impedance-Gradient Absorber: Design and Microwave Absorption Performance
Source: Nanomaterials (Basel). 2025 Dec 29;16(1):42. doi: 10.3390/nano16010042 (PMC12787895; doi:10.3390/nano16010042)
Supplement: Supplementary file 1 [file nanomaterials-16-00042-s001.zip › nanomaterials-4034265-supplementary.pdf]

Supporting Information

Magnetoresponsive Fiber-Reinforced Periodic Impedance-Gradient Absorber： Design and Microwave absorption performance

Yuan Liang[1], Wei Chen[2], Shude Gu[2], Xu Ding[2], Yuping Duan[2]*.

1. International School of Information Science and Engineering, Dalian University of Technology, Dalian 116085, P. R. China.

2. School of Materials Science and Engineering, Dalian University of Technology, Dalian 116085, P. R. China.

Supporting information note S1

1. Asymptotic regulation mechanism of double-gradient electromagnetic parameters

Theoretical basis: According to transmission line theory, the reflection coefficient (Γ) of an electromagnetic wave propagating through a multilayer medium is determined by the gradient variation in impedance across each layer. In this work, a gradient configuration is employed, combining a low-concentration MWCNTs layer (SP1, dielectric loss layer) on the exterior with a high-concentration CIP layer (DS1, magnetic loss layer) on the interior, to achieve asymptotic matching of dielectric permittivity and magnetic permeability.

The structure achieves: High wave transmittance is achieved through the outer SP1 layer containing 0.5% MWCNTs, which facilitates the efficient entry of incident

electromagnetic waves. Simultaneously, the inner DS1 layer, with 83.3% CIP content, provides strong magnetic loss to enhance energy dissipation. Experimental validation (Figure 3c) shows that the G3 configuration (SP1 + DS1) achieves a minimum reflection loss (RL) of –22 dB over the 8.6–26.4 GHz frequency range, confirming the effectiveness of impedance matching theory in guiding gradient absorber design.

2. Geometric Phase Modulation of Tower-Type Superstructural Cells

Theoretical basis: Based on geometrical optics and the principle of multi-path interference, the tower prism structure—with a 70° side inclination angle—enhances multi-reflection attenuation by extending the electromagnetic wave propagation path (up to 1.7λ) and inducing helical wave trajectories (Figure 6d).

Structural realization: The height of the prismatic cells is fixed at 2 mm, while the base width tapers linearly from 2.0 mm at the bottom to 0.9 mm at the top, forming a periodic hexagonal array with 1.5 mm spacing. This design reduces the structural volume to one-quarter of a corresponding homogeneous configuration, yet enhances energy attenuation to 85% (Figure 2b). These results indicate that geometrical phase modulation significantly improves wave absorption efficiency per unit volume.

3. Electric/magnetic Field Distribution and Loss Mechanism: Multiscale Energy Dissipation Synergy

Frequency band division of magneto-electric coupling loss

Rationale: According to Maxwell's system of equations, the frequency dependence of magnetic and dielectric losses are different. The low frequency band (<10 GHz) is

dominated by dielectric loss, while the high frequency band (>10 GHz) is dominated by magnetic loss (Fig. 4a).

Structural realization: the peak magnetic loss of the underlying CIP/PU (DS1) at 10.4 GHz ($\mu''=0.8$) synergizes with the dielectric loss ($\epsilon''=0.3$) of the surface MWCNTs/PU (SP1) to form a broad-band absorption peak (Fig. 3c). The heterogeneous interface triggers strong interfacial polarization (Fig. 4c), further dissipating energy.

4. Wavelength-matching Effect of The FR4 Wave-transparent Layer

Rationale: Optimized transmission of incident waves is achieved through the use of the FR4 layer. Its low dielectric constant minimizes surface reflections, while wavelength matching further enhances the material’s ability to couple incoming electromagnetic energy into the absorber.

Structural realization: The incorporation of a 0.5 mm FR4 layer results in a shift of the absorption peaks to 11.2 GHz (low-frequency region) and 22.8 GHz (high-frequency region), thereby extending the effective absorption bandwidth to 17.8 GHz (Figure 2f). Simulation results confirm that the bimodal absorption behavior observed in the N5 structure validates the applicability of the quarter-wavelength resonance principle (Figure 3e).

5 Phase Modulation and Interference Effects: Active Control of Phase-Cancellation Interference

Structured application of quarter-wavelength interference theory

Theoretical basis: When the material thickness satisfies the quarter-wavelength condition, a phase difference of 180° arises between the reflected waves at the air–absorber interface (Rf) and the absorber–metal backplane interface (Rb), resulting in phase-canceling interference (Figure 5a1).

Structural realization: HFSS simulation of the ASM structure with a total thickness of 3.5 mm (and an effective wave-interacting thickness of 2 mm) at 11.2 GHz reveals that the RL peak aligns with the phase inversion point (Figure 5b1), thereby confirming the predictive validity of the quarter-wavelength theory in guiding structural thickness design.

6 Multilevel Interference Enhancement at Heterogeneous Interfaces

Theoretical basis: The multi-interface design in the ASM structure—comprising the FR4 layer, gradient dielectric layer, and magnetic layer—enhances phase-canceling interference by increasing the number of heterogeneous interfaces (Figure 4c).

Structural realization: Compared to the SAM structure (without FR4), the interference-related attenuation contribution in the ASM structure increases from 45% to 68% (Figure 5a3), indicating that the structured multi-interface design significantly improves broadband absorption performance.

7 Radar Cross-Section and Polarization Properties: Geometrical Symmetry and Scattering Suppression

Polarization-insensitive mechanism with quadruple rotational symmetry

Rationale: According to Jones matrix theory, a structure with fourfold rotational

symmetry (Figure 7e) exhibits polarization-invariant behavior in response to TE and TM polarized waves. As a result, its absorption rate remains stable under varying polarization angles.

Structural realization: The hexagonally periodic arrangement of tower-shaped cells exhibits absorptivity fluctuations of less than 3% under incidence angles ranging from 0° to 60° (Figures 7a–d), along with polarization stability exceeding 94%. These results confirm that the geometric symmetry of the structure effectively modulates its electromagnetic response.

8 RCS Scaling Mechanism of the Tower Structure

Theoretical basis: According to the physical optics model, a prism side inclination angle of 70° induces spiral propagation of electromagnetic waves. This geometric configuration redistributes scattered energy from specular reflection toward fringe bypassing, thereby reducing the far-field radar cross-section (RCS).

Structural realization: Compared with the perfect electric conductor (PEC), the ASM structure reduces the scattering peak at 22.4 GHz (Figure 6c), indicating that its geometric design effectively suppresses radar detection signals by introducing wave path randomization.

This work demonstrates a multiscale effect: macroscopically, the gradient impedance structure enhances the absorption of obliquely incident electromagnetic waves and promotes multiple internal reflections. Microscopically, carbonyl iron and carbon nanotubes exhibit distinct loss characteristics, which enable effective impedance

matching regulation.

In summary, the ASM is designed based on dual gradients—structural gradient and concentration gradient. By optimizing the magneto-electric matching concentration of carbonyl iron and carbon nanotubes, the high-frequency absorption capability is significantly improved. The proposed ASM structure exhibits strong far-field RCS suppression and wide-angle wave-absorbing performance.

Supporting Information Note S2

The SCIP and its dispersion morphology within the polyurethane matrix were characterized using an SU5000 field emission scanning electron microscope (SEM). Additionally, the dielectric constant and magnetic permeability of the ASM structure were measured using an Agilent N5230A vector network analyzer to provide input parameters for electromagnetic simulations.

Reflection loss measurements of the metamaterials were conducted in a microwave anechoic chamber, following the national standard GJB2038A-2011 bow method. Simulations of power loss density distribution and current vector fields were performed using ANSYS HFSS software to elucidate the underlying energy dissipation mechanisms.

Supporting Information Note S3

The simulations were conducted using the finite element–based High Frequency

Structural Simulator (HFSS) software from ANSYS. A metamaterial model with dimensions of 200 × 200 × 3.5 mm was constructed, and the corresponding electromagnetic parameters were assigned accordingly.

The Floquet port excitation and master–slave boundary conditions were applied within a vacuum simulation domain measuring 200 × 200 × 250 mm. Simulations were carried out over the frequency range of 2 to 30 GHz, with a step size of 0.1 GHz. Reflection loss (RL) curves and electromagnetic field distribution profiles were obtained.

Tab. S1 Details of the High Frequency Structure Simulator

Name	Parameter setting	Name	Parameter setting
Base material	Aluminium sheet	Material	SCIP, Aluminium
Material parameters	Fig. 2 (a-h)	Material size	200*200*3.5 mm
Environmental conditions	Vacuum	Environmental scope	200*200*250 mm
Incentive mode	Floquet Port	Analysis method	Sweep
Scanning frequency band	2-30 GHz	Scanning step size	0.1 GHz

Supporting Information Note S4

In this study, a fiber cloth is employed as a structural template, and a wave-absorbing adhesive is applied to its surface to fabricate the prepreg. The dielectric loss mechanism, as illustrated in the ε''–h plot, is primarily attributed to conductive loss and dielectric relaxation, which are influenced by electrical conductivity and molecular polarization, respectively, under varying electromagnetic field conditions.

Variations in the imaginary part of the dielectric constant lead to corresponding changes in its real part, thereby affecting the electromagnetic wave absorption performance. This dynamic may result in impedance mismatch at high frequencies.

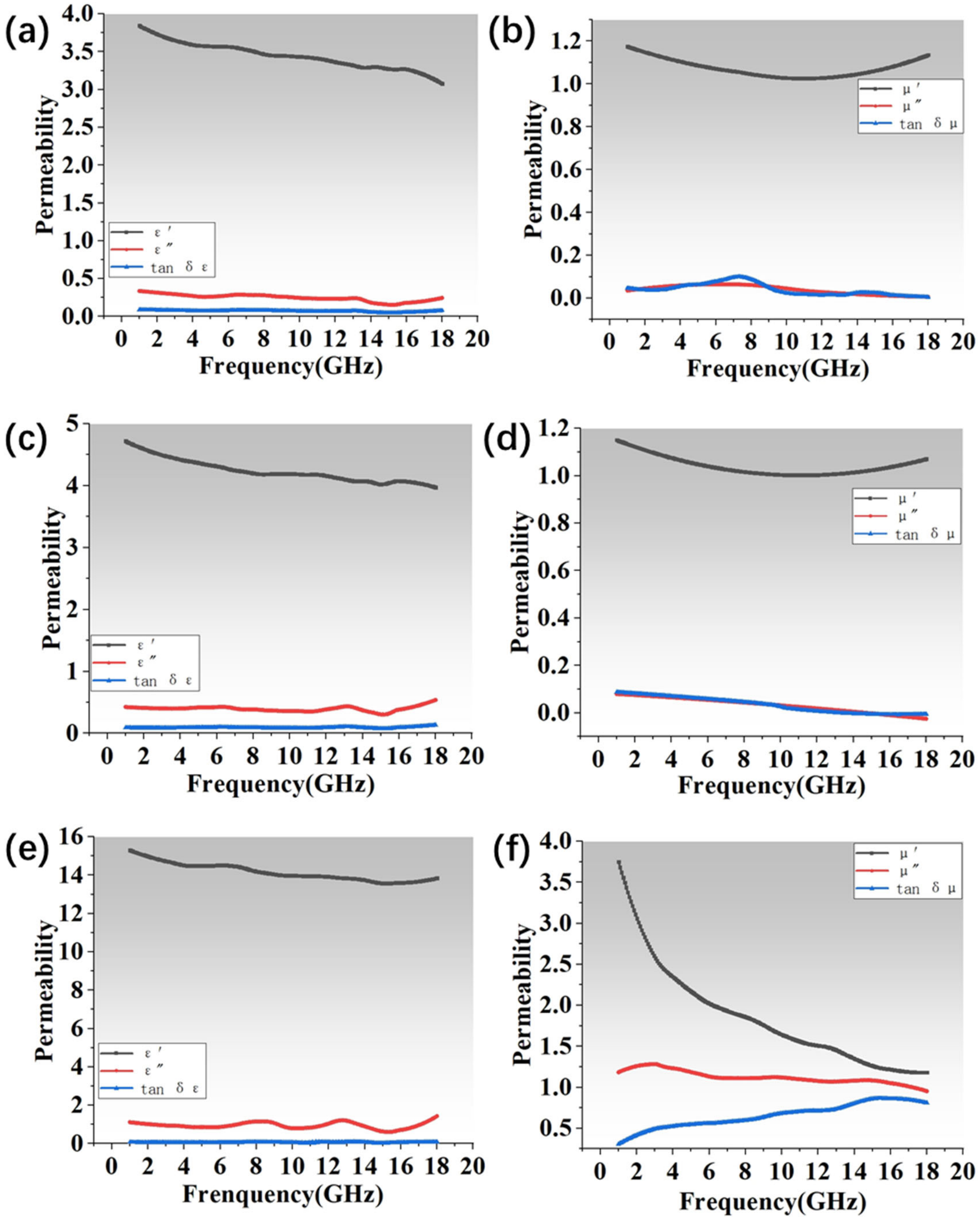
(a)
Permeability
Frequency(GHz)
ε′
ε″
tan δ ε
(b)
Permeability
Frequency(GHz)
μ′
μ″
tan δ μ
(c)
Permeability
Frequency(GHz)
ε′
ε″
tan δ ε
(d)
Permeability
Frequency(GHz)
μ′
μ″
tan δ μ
(e)
Permeability
Frenquency(GHz)
ε′
ε″
tan δ ε
(f)
Permeability
Frequency(GHz)
μ′
μ″
tan δ μ

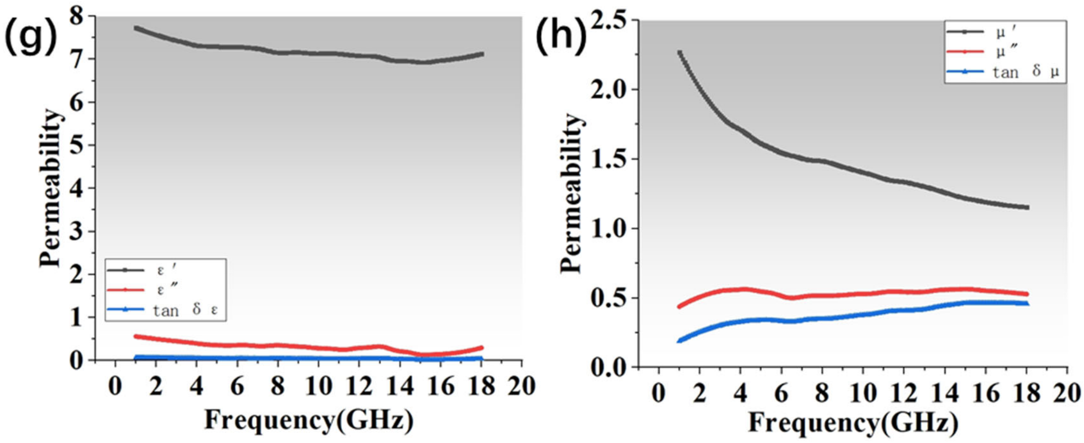

Fig. S1 (a, b) SP1 dielectric constant versus magnetic permeability. (c, d) SP2 permittivity versus magnetic permeability. (e, f) DS1 permittivity vs. permeability. (g, h) DS2 permittivity vs. magnetic permeability.

Supporting Information Note S5

Tab. S2 SCIP mass, PU mass, surface density, and bulk density for different proportions of ASM

SCIP:PU	m_{SCIP}(g)	m_{PU}(g)	Surface density (g/cm^2)	Bulk density (g/cm^3)
1:1	75.96	75.96	0.38	1.89
2:1	135.55	67.77	0.51	2.54
3:1	183.54	61.18	0.61	3.05
4:1	223.02	55.76	0.70	3.48
5:1	256.07	51.21	0.77	3.84

Supporting information note S6

ASM Performance Advantage Comparison

absorber	EAB(GHz)	Thicknesses (mm)	Bibliography
CIP/MWCNTs	18.2	3.5	This Work
Si_3N_4	4.2	3.75	[1]

$Fe_3O_{4/}$ CNT	8.3	3	[2]
MWCNTs	11.3	8	[3]
CIP/Cf	21.7	8	[4]
Cu-Co	6.8	2.8	[5]
Si_3N_4	4.2	3.75	[6]
SCI/MWCNT	12.3	7.59	[7]

References

[1] F. Ye, Q. Song, Z. Zhang, W. Li, S. Zhang, X Yin, Y Zhou，H. Tao, Y. Liu, L. Cheng , Adv. Funct. Mater. 2018, 28, 1707205.

[2] N. Li, Huang, G. W. Huang. Y. Q. Li, H. M. Xiao, Q. P. Feng, N. Hu, S. Y. Fu, ACS Appl. Mater. Interfaces. 2017, 9 (3), 2973−2983.

[3] Q. Zhou, X.W. Yin, F. Ye, X.F. Liu, L.F. Cheng, L.T. Zhang, Mater. Des. 2017, 123, 46-53.

[4] P. F. Yin, G. L. Wu, Y. T. Tang, Chem. Eng. J. 2022, 446, 136975.

[5] J. Liu, L. Zhang, D. Zang, H. Wu, Adv. Funct. Mater. 2021, 31, 2105018

[6] F. Ye, Q. Song, Z. Zhang, W. Li, S. Zhang, X Yin, Y Zhou，H. Tao, Y. Liu, L. Cheng , Adv. Funct. Mater. 2018, 28, 1707205.

[7] Y. Huang, X. Yuan, M. Chen, W. L. Song，D. Fang, ACS Appl. Mater. Interfaces. 2018, 10, 44731－44740.
